# Signalling-Dependent Adverse Health Effects of Carbon Nanoparticles Are Prevented by the Compatible Solute Mannosylglycerate (Firoin) *In Vitro* and *In Vivo*

**DOI:** 10.1371/journal.pone.0111485

**Published:** 2014-11-21

**Authors:** Andrea Autengruber, Ulrich Sydlik, Matthias Kroker, Tamara Hornstein, Niloofar Ale-Agha, Daniel Stöckmann, Andreas Bilstein, Catrin Albrecht, Adnana Paunel-Görgülü, Christoph V. Suschek, Jean Krutmann, Klaus Unfried

**Affiliations:** 1 IUF Leibniz Research Institute for Environmental Medicine, Düsseldorf, Germany; 2 bitop AG, Witten, Germany; 3 Department of Traumatology and Hand Surgery, Heinrich-Heine-University of Düsseldorf, Düsseldorf, Germany; 4 Zentrum für Molekulare Medizin der Medizinischen Fakultät der Heinrich-Heine-Universität, Düsseldorf, Germany; National Institutes of Health (NIH), United States of America

## Abstract

The inhalation of combustion-derived nanoparticles leads to adverse health effects in the airways. In this context the induction of membrane-coupled signalling is considered as causative for changes in tissue homeostasis and pro-inflammatory reactions. The identification of these molecular cell reactions allowed to seek for strategies which interfere with these adverse effects. In the current study, we investigated the structurally different compatible solutes mannosylglycerate (firoin) from thermophilic bacteria and ectoine from halophilic bacteria for their capability to reduce signalling pathways triggered by carbon nanoparticles in target cells in the lung. The pre-treatment of lung epithelial cells with both substances decreased the particle-specific activation of mitogen-activated protein kinases and also the endpoints proliferation and apoptosis. Firoin applied into the lungs of animals, like ectoine, led to a significant reduction of the neutrophilic lung inflammation induced by particle exposure. The pro-inflammatory effect of carbon nanoparticles on human neutrophil granulocytes ex vivo was significantly reduced by both substances via the reduction of the anti-apoptotic membrane-dependent signalling. The data of this study together with earlier studies demonstrate that two structurally non-related compatible solutes are able to prevent pathogenic reactions of the airways to carbon nanoparticles by interfering with signalling events. The findings highlight the preventive or therapeutic potential of compatible solutes for adverse health effects caused by particle exposure of the airways.

## Introduction

The inhalation of combustion-derived carbonaceous nanoparticles leads to adverse health effects in the airways including lung cancer, chronic obstructive pulmonary disease, and fibrosis [1]–[4]. The induction of membrane coupled signalling pathways in lung epithelial cells and immune cells residing in the respiratory tract has been identified to be critical for the toxic potential of these xenobiotics. In lung epithelial cells, this interaction is governed by the ligand-independent activation of the epidermal growth factor receptor (EGFR) leading to the activation of signalling pathways including mitogen-activated protein kinases (MAPK) [5]–[7]. In studies with human and rat lung epithelial cells we found that pure carbon nanoparticles (CNP) induced adverse endpoints like the up-regulation of pro-inflammatory cytokines, apoptosis and proliferation [5],[8]. These endpoints are mediated by separate specific MAPK pathways, while Erk1/2 activation is a pre-requisite for proliferation, the phosphorylation of JNK1/2 is specific for CNP-triggered apoptosis. CNP-specific activation of the MAPK p38 leads to an up-regulation and increased release of the pro-inflammatory chemokine IL-8 [8]. In neutrophilic granulocytes, CNP trigger specific membrane-dependent signalling pathways which reduce the natural apoptosis rates of these immune cells and therefore contribute to the aggravation of the inflammation [9].

During the last years, we aimed to develop preventive strategies based on the knowledge of these molecular events which occur during the interaction of environmentally relevant combustion-derived nanoparticles and lung epithelial cells. For that purpose, we screened substances which are well tolerated by epithelial cells for their ability to prevent membrane-coupled cell stress events triggered by carbon nanoparticles. We identified the ‘compatible solute’ ectoine as effective in preventing cell stress reactions triggered by carbon nanoparticles. Ectoine prevented the downstream consequences of the specific changes in the lipid raft induced by nanoparticles [7]. As the main outcome of this preventive action, MAPK-dependent neutrophilic inflammation in animals was significantly reduced when CNP were administered in the presence of ectoine [8]. More recently, we were able to show that CNP in an ongoing inflammation reduce the natural apoptosis of neutrophils and therefore contribute to the aggravation of the inflammation. In this scenario, the application of ectoine restored natural apoptosis rates by preventing adverse membrane-coupled signalling in neutrophils and thereby led to a reduction of neutrophilic lung inflammation [9].

Compatible solutes are defined as small, organic, mostly neutral or zwitter-ionic compounds, which are able to stabilize cell functions [10]. The capability to stabilize macromolecules by influencing the availability of water molecules in the hydration layer (preferential exclusion) is considered as the main mechanism by which these substances interact with biological systems [11]. The beneficial effects on molecules have been impressively demonstrated by mechanistic approaches studying the unfolding of membrane proteins or the fluidity of artificial membranes [12]–[14]. These properties make them ideal candidates for biotechnological applications [15]. But also medical applications and therapeutic approaches in which stabilization of cellular structures and functions are considered to be beneficial have been suggested and were successfully tested in pre-clinical and clinical studies [16],[17]. Although compatible solutes are found in almost all living organisms, in particular extremophilic bacteria are important sources for these substances. These microorganisms synthesize high amounts of the substances in order to prevent cell stress in their natural ecosystems. Ectoine (1,4,5,6-tetrahydro-2-methyl-4-pyrimidinecarboxylic acid) for example is produced by halophilic bacteria as an osmolyte counteracting osmotic stress [18]. Firoin (mannosyglycerate) is found in thermophilic bacteria like *Pyrococcus furiosus* and has been described to be an effective stabilizer of proteins under temperature stress [19]–[21].

In the present study we tested the preventive effects of the compatible solutes ectoine and firoin on particle-exposed lung epithelial cells. The investigations of ectoine and firoin as examples of compatible solutes coming from halophilic and thermophilic bacteria were performed as a proof of concept for the strategy to use different kinds of compatible solutes as preventive substances in the airways. Comparing two chemically independent substances with respect to their efficacy might provide valuable information for further efforts to find substances for molecular prevention. Both substances were tested for their capacity to prevent CNP-induced apoptosis and proliferation and the respective signalling events specifically triggered by environmental model particles in lung epithelial cells. Furthermore, the effects of firoin on lung inflammation in the in vivo system was studied. The possible value of firoin on the prevention of anti-apoptotic effects was investigated in human peripheral blood neutrophils. The efficacy of firoin in the in vivo and the ex vivo systems was compared with the previously published preventive effects of ectoine.

## Methods

### Ethics statement

The human study was approved by the local ethics committee on human research of the Heinrich-Heine University Düsseldorf and written informed consent was obtained from all study subjects before enrolment. Animal studies according to German animal welfare legislation were approved by the responsible authority (LANUV NRW).

### Reagents

CNP, 14 nm diameter (Carbon Black, Printex 90, Degussa, Frankfurt, Germany) were characterized and prepared as described earlier [5],[7]. Ectoine ((S)-2-methyl-1,4,5,6-tetrahydropyrimidine-4-carboxylic acid, LPS-free, ultrapure 99%, bitop AG, Witten, Germany) and firoin mannosylglycerate, 99% bitop AG, Witten, Germany) were solubilized in PBS. Possible endotoxin contaminations were counteracted by purifying solubilized substances using endotoxin removal spin columns from Thermo Scientific (Germany).

### Cell Culture Experiments

RLE-6TN cells [22] (ATCC, Manassas, VA) were cultured as described earlier [5]. Cells were treated with particles for 4 h (JNK1/2, caspase activity), 8 h (Erk1/2), or 24 h (BrdU incorporation) in the absence or presence of compatible solutes. Compatible solutes were added 1 h prior to particle exposure to achieve final concentrations of 5 mM, 1 mM, 0.1 mM, and 0.001 mM for the signalling experiments. The final concentrations for apoptosis were 1 mM (ectoine) and 5 mM (firoin) and for proliferation 1 mM for both substances. The incorporation of 5-bromo-2′-deoxy-uridine (BrdU) was monitored using the BrdU Labeling and Detection Kit III (Roche Applied Science, Mannheim, Germany). Caspase-3 activity was determined using Caspase-3 Assay Kit (BD Pharmingen, San Diego, CA).

### Protein Analyses

Protein isolation and Western blotting were performed as described earlier [5],[9],[23] using antibodies specific for phospho-p44/42 MAPK (Erk1/2) (Thr202/Tyr204), and phospho-SAPK/JNK (Thr183/Tyr185). Total ERK1/2, JNK1/2, and GAPDH (Imgenex, San Diego, CA) proteins were monitored with p44/42 MAPK and SAPK/JNK antibodies, respectively. Unless otherwise stated, all antibodies were from Cell Signaling Technology (Danvers, MA). Human Mcl-1 was detected using an antibody from BD Pharmingen (San Jose, CA). Signal strength was detected using the ECL method. All Western blot results were quantified densitometrically. Cinc-1 levels were detected by rat cytokine antibody arrays (RayBiotech, Inc., Norcross, CA) according to manufacturers' instructions.

### Animal Experiments (Experimental Design)

Female Fisher 344 rats (8 weeks old, Charles River Laboratories, Germany) were instilled intratracheally with 2.5 mg/kg CNP suspended in 0.4 ml PBS as described earlier [8]. After 48 hours, BAL and lung samples were taken. Differential cell counts were performed from Giemsa/May-Grünwald staining of lavage cells. Cell-free lavage fluids were used for Cinc-1 assays. Lung tissues were either minced, shock frozen and stored at −80°C or infused with paraformaldehyde fixing solution, dehydrated and embedded in paraffin. All animal experiments were performed after relevant permission according to German animal protection laws.

### Lung Histology

Tissue preparation, sections, and immunohistochemical staining were performed as described [24]. In short, 2 µm paraffin-embedded sections were re-hydrated, epitopes were unmasked with citrate buffer and treated with H_2_O_2_. After block with goat serum, sections were stained over night with phospho-Erk1/2 or phospho-JNK1/2 using the corresponding Western blot antibodies and detected with HRP-coupled secondary antibody and AEC substrate and counterstained with hematoxylin. Neutrophil elastase was stained accordingly with the rat specific antibody (Abcam, Cambridge, USA). The specific staining was performed using diaminobenzidine. The specificity of the staining was controlled by parallel approaches without primary antibody and with isotype controls.

### Isolation of Human Neutrophilic Granulocytes and Neutrophil Apoptosis

Peripheral blood was collected from young, healthy, male volunteers (age 31.2±7.3 yrs). Volunteers with ongoing medication were not included in the study. Neutrophil isolation was performed as described [23]. Firoin (1 mM in PBS) was added to the neutrophilic cell culture 2 h prior to particle treatment (33 µg/ml in PBS). Cells were harvested after 6 h (signalling proteins) and 16 h (apoptosis). Apoptosis was analysed using Annexin V Staining Detection kit from eBioscience (San Diego, CA). Fluorescence was measured flow cytometrically (BD FACS Canto) by counting a minimum of 10^4^ events per sample and positive cells were considered as apoptotic. Different treatment groups were normalized to spontaneous apoptosis of untreated cells.

### Statistics

Statistical analyses were performed using IBM SPSS statistics 22. Figures display mean values and standard errors. As indicated in the figure legends, different kinds of analyses were performed. Normally distributed samples were analysed by one-way ANOVA with the indicated post hoc significance testing. Small sample size groups were compared employing the non-parametric Mann-Whitney-U test. Corrections for multiple testing were performed when applicable.

## Results

### Firoin and Ectoine Prevent CNP-dependent MAPK Activation in Lung Epithelial Cells

**Figure 1:**
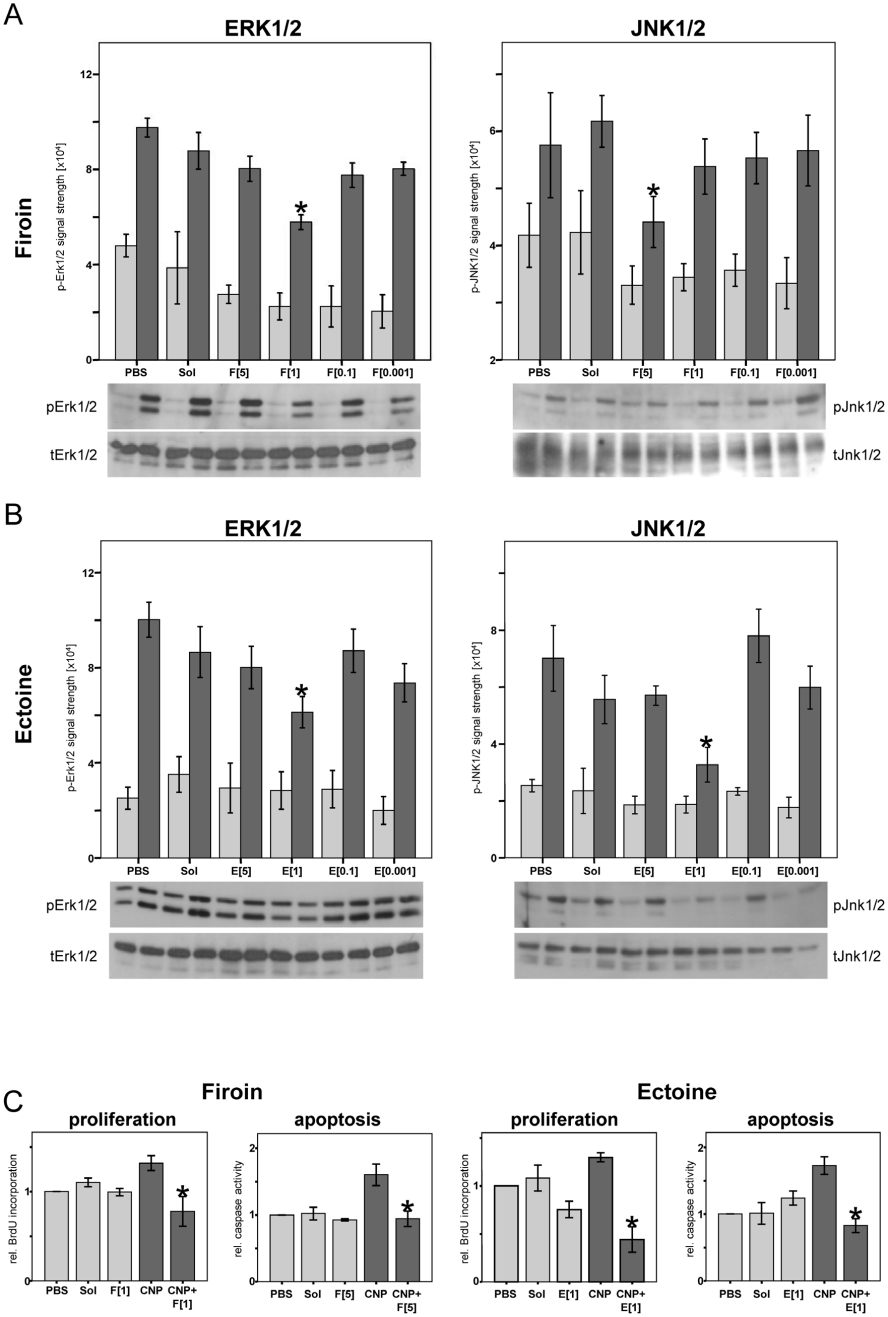
Firoin and Ectoine Prevent CNP-induced MAPK Activation and Subsequent Endpoints of Tissue Homeostasis. RLE cells were pre-treated with the indicated final concentrations [mM] of firoin *(F)* in (A), ectoine *(E)* in (B), or controls PBS or H_2_O (Sol), 1 h prior to CNP exposure. Quantitative analysis of MAPK phosphorylation (n = 3–5) and representative Western-blots. Light bars, control treatment with PBS, dark bars, CNP-treatment [10 µg/cm^2^] for 8 h (Erk1/2) or 4 h (JNK1/2). C: BrdU incorporation and caspase-3 activity (each n = 3) are shown relative to PBS treated controls. Cells were treated as described above. BrdU incorporation was determined after 24 h of exposure, caspase activity after 8 h. * Significantly different from CNP alone exposed controls (p<0.05 ANOVA and Tukey-HSD post hoc testing).

In a first approach to investigate the beneficial effects of the compatible solutes ectoine and firoin, the phosphorylating activation of the MAPK Erk1/2 and JNK1/2 was investigated in the cell culture system with rat lung epithelial cells (RLE-6TN). Both markers in earlier studies proved to be specific for CNP, as bigger non-nanoparticles did not trigger these cell reactions [5]. Moreover, the specific link of these pathways to the endpoints proliferation (Erk1/2) and apoptosis (JNK1/2) has been demonstrated by specific pharmacological intervention [5]. Rat lung epithelial cells were either pre-treated with the compatible solutes for 1 h with the indicated concentrations of the substances or mock treated. After exposure to CNP [10 µg/cm^2^], the phosphorylation of the MAPK was determined at the time point of maximal activation (4 h for JNK1/2, 8 h for Erk1/2). This particle exposure conditions were chosen from earlier dose response experiments on MAPK activation in which no cytotoxic side effects were observed [5]. The activation of MAPK was measured in Western blots by detecting the amount of phosphorylated protein while the total protein content of the respective MAPK remained unchanged. The pre-treatment with the compatible solutes alone in none of the experiments led to a significant change in phosphorylation rates (figure 1). The induction of the signalling events triggered by the CNP treatment, however was reduced by the compatible solutes. For both substances and both endpoints these effects occurred in a rather narrow concentration range. The presence of 1 mM firoin was sufficient to prevent Erk1/2 activation after particle treatment but for the reduction of phosphorylated JNK1/2 a final concentration of 5 mM firoin was necessary (figure 1A). However, 1 mM ectoine led to a significant reduction of phosphorylation of both MAPK, Erk1/2 and JNK1/2 (figure 1B).

**Figure 2:**
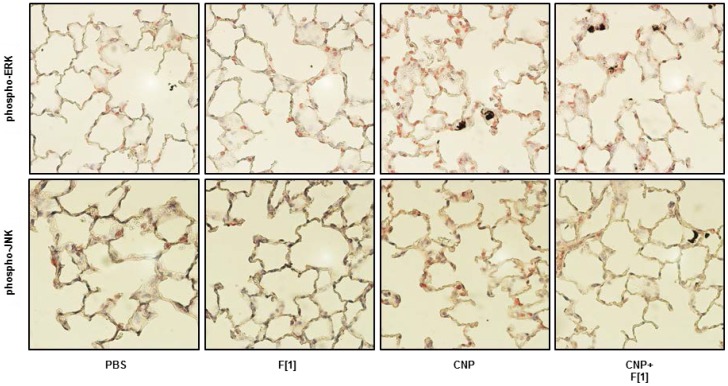
Particle-induced Activation of MAPK Is Blocked by Firoin. Female Fischer 344 rats were exposed to 2.5 mg/kg CNP in the presence or absence of 1 mM firoin (see fig. 3A). Phoshpo-Erk1/2 and phospho-JNK1/2 signals were stained (red) in 2 µm paraffin-embedded lung sections taken from animals 48 h after exposure.

**Figure 3:**
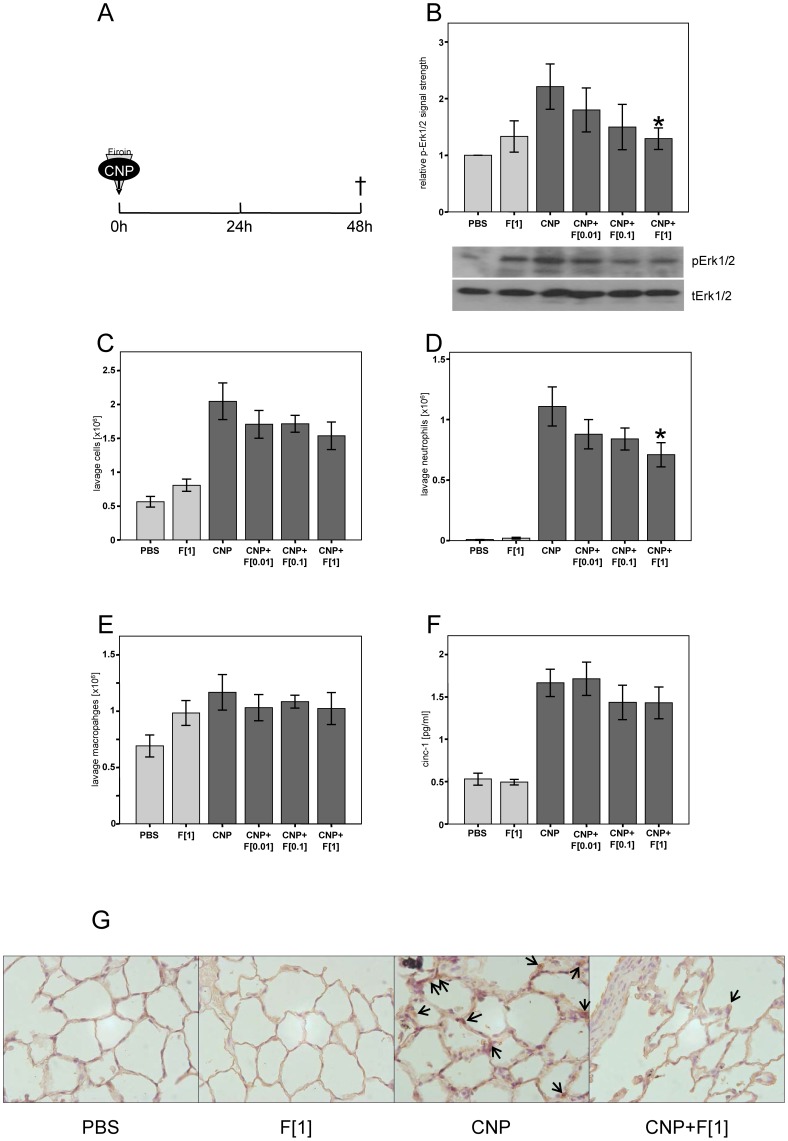
Firoin Reduces MAPK Activation and Neutrophilic Lung Inflammation in vivo. Female Fischer 344 rats (n = 7) were exposed to 2.5 mg/kg CNP in the presence or absence of the indicated doses [mM] of firoin *(F)*. A: exposure scenario. B: Quantification of phospho-specific signals in lung homogenates in relation to total Erk1/2 and representative Western blots. Total cell counts (C), numbers of neutrophils (D) and macrophages (E), and cinc-1 concentrations (F) were determined in bronchoalveolar lavage. (G) Staining of neutrophil elastase in lungs of animals treated according to figure 3A. Light bars, control groups with PBS or 1 mM firoin exposure; dark bars, CNP exposed animals. * Significantly different from CNP alone treated animals (p<0.05, Mann-Whitney-U Test). Arrows indicate cells considered as positive for neutrophil elastase.

The functional relevance of the prevention of cell stress signalling was also tested by measuring the respective endpoints specifically mediated in lung epithelial cells by these MAPK. For this purpose, the most efficient compatible solute concentrations which inhibited phosphorylation of Erk1/2 and JNK1/2 were used to interfere with the cascade leading to proliferation and apoptosis induced by CNP treatment. BrdU incorporation and activation of caspase-3 were measured in RLE cells, respectively. In accordance with the MAPK findings, the incorporation of BrdU as well as the activation of caspase-3 triggered by carbon nanoparticle stress was significantly reduced when the cells were pre-exposed to the compatible solutes (figure 1C). The in vivo relevance of the activation of MAPK by CNP and also the potential of firoin to prevent this cell stress reaction was tested by immunostaining of lung sections from rats exposed to CNP in the presence and absence of this substance (figure 2). The activation levels of both MAPK were elevated in the lung epithelium 48 h after CNP exposure. The application of firoin together with the particles (experimental design, figure 3A) was able to prevent these cell reactions.

### Firoin Prevents CNP-induced Neutrophilic Lung Inflammation

As a major outcome of CNP exposure, lung inflammation was investigated for its sensitivity to firoin treatment. In a first approach, increasing doses of the substance were applied together with the particles in rats (figure 3A). The membrane-dependent activation of Erk1/2 which was earlier correlated with the induction of neutrophilic lung inflammation in vivo [7],[8] was determined by Western blot analyses. After 48 h, a reduction of Erk1/2 phosphorylation by increasing concentrations of firoin was observed. Firoin treatment also resulted in an attenuation of the inflammation due to the significant reduction of neutrophils in bronchoalveolar lavage (BAL), compared to animals treated with CNP alone (figure 3D). The number of macrophages remained unchanged by the firoin application. The effect of firoin was also observed at the level of total lavage cell number as well as the neutrophil-recruiting chemokine cinc-1, however these effects proved not to be statistically significant (figure 3C, 3F). The reduction of neutrophilic lung inflammation was also visualised at the level of interstitial and adherent neutrophils by immunostaining of neutrophil elastase in lung tissue from animals which were exposed for 48 h (figure 3G).

**Figure 4:**
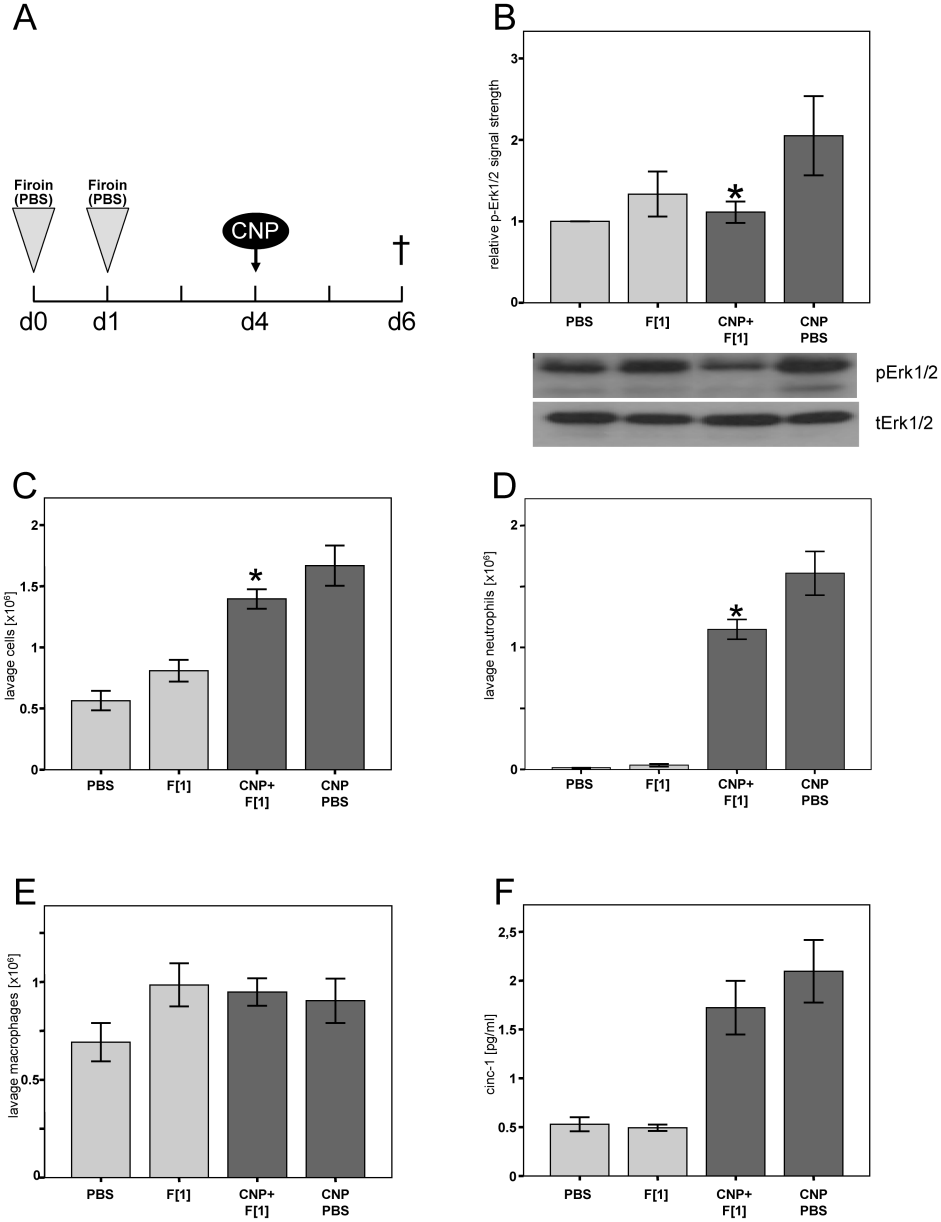
Firoin Acts in a Preventive Manner. Female Fischer 344 (n = 7) rats were pre-treated with 1 mM firoin *(F)* and subsequently exposed to 2.5 mg/kg CNP as depicted in A. B: Quantification of phospho-specific signals in relation to total Erk1/2 and representative Western blots. Total cell counts (C), numbers of neutrophils (D) and macrophages (E), and cinc-1 concentrations (F) were determined. Light bars, control groups with PBS or firoin pre-treatment; dark bars, PBS or firoin pre-treated animals exposed to CNP. * Significantly different from PBS pre-treated CNP-exposed animals (p<0.05, Mann-Whitney-U Test).

Although compatible solutes, as hydrophilic substances, are not considered to interact with the hydrophobic carbon particles, they might influence particle characteristics in a way that less pro-inflammatory events are triggered after particle-cell interaction. Therefore, as a proof of principle, the application of firoin was separated from the application of CNP. Animals were treated twice with firoin prior to the exposure to CNP (figure 4A). Again, the phosphorylation of Erk1/2 in lung homogenates was significantly less pronounced in firoin pre-treated animals compared to animals pre-treated with PBS alone (figure 4B). Accordingly, neutrophilic lung inflammation at the level of total BAL cell numbers and neutrophils was reduced by the preventive treatment (figure 4C, 4D), while the number of macrophages remained unchanged (figure 4E).

### Effect of Firoin on Human Neutrophil Apoptosis

**Figure 5:**
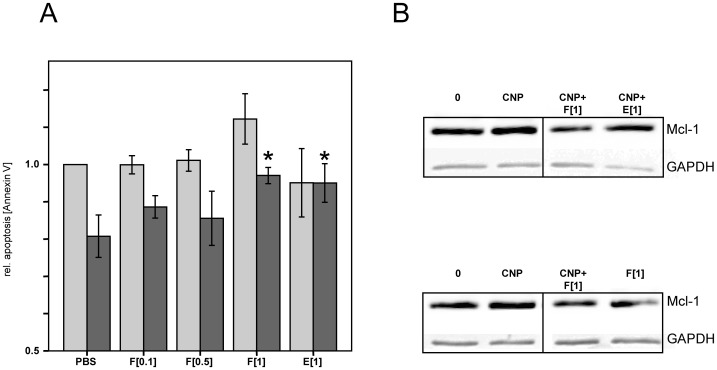
Particle-induced Changes in Apoptosis are Restored by Firoin. Peripheral blood neutrophils from healthy donors (n = 7), 2 h pre-treated with indicated amounts of firoin *(F)* or 1 mM ectoine *(E)* before CNP exposition (33 µg/ml). A: After 16 h of CNP treatment, cells were stained with Annexin V and analysed flow cytometrically. Normalized values of % Annexin V-positive cells are shown. Natural apoptosis of untreated cells was considered as 1. Dark bars, CNP-treated, light bars, untreated. * Significantly different from CNP alone (Mann-Whitney U Test after Bonferroni correction for multiple testing p<0.05). B: 6 h post CNP-treatment the anti-apoptotic Mcl-1 expression was measured by Western blot analysis. GAPDH was used as a loading control. Two representative blots from different individuals are shown and irrelevant lanes were removed.

Previous studies identified the role of ectoine not only in reducing the pro-inflammatory response of epithelial cells to CNP, but also to restore normal levels of apoptosis in CNP-treated neutrophils. To evaluate whether firoin also has a beneficial effect on this pro-inflammatory event, human peripheral blood granulocytes from healthy volunteers (n = 7) were pre-treated ex vivo with increasing doses of firoin 2 h prior to CNP exposure [33 µg/ml]. Cells were analysed after 6 h of particle exposure for the expression of anti-apoptotic Mcl-1 protein and apoptosis rates were determined flow cytometrically after 16 h using the early apoptotic marker Annexin V. As an internal control, the experiment was also performed in the presence of 1 mM ectoine. Pre-treatment of 1 mM firoin significantly restored natural apoptosis rates which were reduced by the exposure of the neutrophils to CNP (figure 5A). The same effect was observed for the compatible solute ectoine. Although statistically not significant, it appears that firoin in contrast to ectoine slightly increases natural apoptosis in the absence of CNP. The preventive effect of firoin and ectoine was also obvious at the level of the anti-apoptotic protein Mcl-1. While treatment of neutrophils with CNP increased Mcl-1 protein amounts, this effect was attenuated in cells pre-treated with the compatible solutes, as shown by two representative Western blots (figure 5B).

## Discussion

With the current findings we describe preventive effects of two structurally unrelated compatible solutes on membrane dependent signalling pathways induced by carbon nanoparticles. The major results are: (i) the pre-treatment of lung epithelial cells with firoin and ectoine leads to reduced CNP-specific MAPK activation responsible for aberrant proliferation or apoptosis (ii) the application of firoin as earlier demonstrated for ectoine, has preventive effects on neutrophilic lung inflammation (iii) in the presence of firoin and ectoine, the anti-apoptotic effects of CNP on peripheral human neutrophils are prevented and natural apoptosis rates are restored.

### Effects of Compatible Solutes on MAPK Activation, Proliferation, and Apoptosis

MAPK activation by CNP as model particles for ultrafine ambient air particles can be specifically and dose dependently reduced by the application of both compatible solutes. Accordingly, proliferation and apoptosis which both occur in cultured lung epithelial cells after CNP exposure depending on the pre-disposition of the individual cell were significantly reduced in these experiments. The reduction of proliferation and apoptosis demonstrate the physiological relevance of the signalling pathways via Erk1/2 and JNK1/2 for these in vitro endpoints. The findings corroborate the causal link between the signalling pathways activated by CNP and the specific outcomes.

Besides possible effects on tissue homeostasis and remodelling, the most prominent effect of inhaled ambient particles is the induction of inflammatory responses. The recent data show that both markers of membrane-dependent signalling are induced in the lung epithelium also in vivo by CNP and that the prevention of this cellular reaction by an intervention strategy can be correlated to a reduction of neutrophilic inflammation in rat lung. The link between MAPK activation and the expression of neutrophil-attracting chemokines has earlier been demonstrated in a study in which human bronchial epithelial cells have been exposed to CNP [25]. These data demonstrate that the redox-dependent activation of MAPK in response to CNP in epithelial cells rather than the initiation of the NFκ-B pathway is of relevance for the pro-inflammatory reaction of the lung. The activation of MAPK in lung epithelial cells can therefore be an indicator of the pro-inflammatory potential of untested materials like engineered nanoparticles and might be useful for toxicity testing of such materials. Furthermore, the in vitro system of the activation of MAPK together with the endpoints proliferation and apoptosis induced by CNP could be a tool to screen substances for their potential to prevent cell stress reactions at the membrane level.

### Effects of Compatible Solutes on Neutrophilic Lung Inflammation in Rats

Applying firoin either together with or prior to the particles into the lungs of animals resulted in a mild (20% to 30%) but significant reduction of neutrophilic lung inflammation 48 h after particle exposure. This effect, however, is stronger in other experimental systems. We recently demonstrated an ectoine-caused reduction of the inflammatory response by 50% in CNP-treated C57/Bl6 mice 12 h after particle exposure [26]. The efficacy of firoin to reduce neutrophilic cell numbers in the rat system was in about the same range as the same dose of ectoine, as we observed earlier also in rats [8]. In these studies, we also performed control experiments testing urea as a solute which is not considered as 'compatible'. This substance is not able to stabilize macromolecules according to the principle of preferential exclusion and therefore had no beneficial effect on CNP-induced lung inflammation.

The success of the strategy to apply the compatible solute prior to the particle exposure underlines the preventive way of action of compatible solutes, mediated by the stabilization of the membrane-coupled signalling complexes. However, from the animal experiments it cannot be concluded that the preventive effect of firoin on the lung inflammation is exclusively mediated by the reduction of pro-inflammatory reactions of the epithelium. Although not studied at a functional level it is evident, that in both animal experiments firoin treatment has no effect on the number of macrophages in the lung at the time point of analysis. This can partially be explained by the observed predominant effect of compatible solutes on the expression of the neutrophil recruiting chemokine cinc-1 in epithelial cells. On the other hand, a direct effect of firoin on neutrophils cannot be excluded.

### Effects of Compatible Solutes on Neutrophil Life Span

We earlier described that during an ongoing inflammation, CNP in neutrophils counteract the natural apoptosis. Apoptosis of neutrophils is an important regulatory mechanism of an acute inflammation [27]. The natural life span of fully differentiated neutrophils can be prolonged in the presence of inflammatory mediators or environmental pollution like CNP by stimulating anti-apoptotic signalling. In the case of a chronic inflammation, however, this reaction is detrimental as the destructive potential of activated neutrophils on the tissue is increased. The restoration of the natural apoptosis rates due to the application of ectoine has been shown to contribute to the resolution of the inflammation in the animal system even after repetitive particle exposure [9]. In particular, in degenerative lung diseases like chronic obstructive pulmonary diseases (COPD) a prevention of these anti-apoptotic events is desirable and might contribute to the reduction or resolution of a chronic lung inflammation. The pre-treatment of human peripheral blood neutrophils as an ex vivo model to study cellular life span with increasing doses of firoin led to a restoration of the natural apoptosis rate which was strongly reduced by CNP exposure. The same effect was observed when the cells were treated in the presence of 1 mM ectoine, as a positive control. We therefore earlier suggested to test ectoine for beneficial effects on chronic lung inflammation in humans [9]. Although statistically not significant, firoin appears to increase apoptosis rates in neutrophils which were not exposed to CNP. This effect was never observed with ectoine and might therefore be a specific feature of firoin which needs to be further investigated.

### The Efficacy of Firoin and Ectoine

Studies comparing chemically different compatible solutes revealed different specific effectivity with respect to their stabilizing capacity on proteins in vitro [28],[29]. Firoin as a substance coming from thermophilic bacteria compared to other compatible solutes, including ectoine, exhibited a high potential to stabilize proteins under thermal stress. The current study compared the efficacy of ectoine and firoin with respect to their preventive capacity in living systems. Except for some minor differences in the cell culture experiments, both substances were able to reduce the adverse effect with almost similar efficacy. This is particularly obvious when our previous data on the effectivity of ectoine to reduce lung inflammation in the animal system are considered [8],[9]. Similar molarities of both substances lead to comparable effects on CNP-induced lung inflammation in rats and to the rescue of the apoptosis rates in human neutrophils. The data therefore confirm the preventive effects of compatible solutes independent of the biological function in the producing organism. We suggest to test a bigger number of compatible solutes for their preventive function and possible therapeutic value in the airways.

We are aware that the main strategy to prevent adverse effects of air pollution has to be the improvement of air quality. However, modern life style as well as non-anthropogenic sources of air pollution often set limits to measures of air hygiene [1]. Due to the increasing use of nanomaterials, additional occupational or environmental exposure scenarios might occur in the future and therefore the development of a molecular preventive approach could be valuable. Additionally, due to pre-existing diseases of the airways, pre-disposed persons may need additional strategies to prevent the adverse effects of inhaled particles. The identification of well tolerated substances like compatible solutes which diminish particle-induced signalling events would be a useful strategy of molecular prevention or in the case of pre-disposed patients a possible support of current therapies.

In conclusion, the data of the current study show that firoin, like ectoine, is a compatible solute which has beneficial effects on adverse effects in the airways after exposure to combustion-derived nanoparticles. Both substances which are chemically and structurally not related have very similar efficacy in the prevention of pathogenic endpoints induced by carbon nanoparticles in vitro and in vivo.
